# Behçet’s Disease in Saudi Arabia: Clinical and Demographic Characteristics

**DOI:** 10.7759/cureus.34867

**Published:** 2023-02-11

**Authors:** Fayez Alharthy, Ahmed S Almaqati, Sarah Alsulami, Akram Ahmad

**Affiliations:** 1 Medicine/Rheumatology, King Saud Bin Abdulaziz University for Health Sciences College of Medicine, Jeddah, SAU; 2 Medicine/Rheumatology, King Abdullah International Medical Research Center, Jeddah, SAU; 3 Medicine/Rheumatology, King Abdulaziz Medical City, Jeddah, SAU; 4 Rheumatology, Al-Noor Specialist Hospital, Mecca, SAU; 5 Cardiology, Dr. Samir Abbas Hospital, Jeddah, SAU

**Keywords:** behcet disease, behcet’s syndrome, anti-tnf agents, neuro-behçet's disease, demographic characteristics

## Abstract

Objective

This study aimed to analyze and determine the clinical features and characteristics of patients diagnosed with Behçet’s disease (BD) in Saudi Arabia.

Methods

This single-center study was conducted in a tertiary care center in the western region of Saudi Arabia. Electronic medical records of patients with BD aged 14 years and older were reviewed and their demographic and clinical data were collected by trained rheumatologists. Between comparisons, Fisher’s exact test, independent t-test, or Mann-Whitney U test was applied. The normality of variables was tested using the Kolmogorov-Smirnov and Shapiro-Wilk tests.

Results

The mean age of symptom onset was 29.6 ± 11.4 years, and mean age at the time of diagnosis was 31.1 ± 11.9 years. Most patients were overweight (mean body mass index 26.7 ± 5.60 kg/m2). The most associated medical comorbidities were diabetes mellitus and hypertension. The most common clinical manifestations were oral ulcers (91.2%), genital ulcers (81.3%), arthritis (41.8%), and pseudofolliculitis (34.1%). Colchicine was the most prescribed treatment (95.6%), followed by prednisolone (72.5%), and azathioprine (36.3%). Male patients were significantly more likely to have pseudofolliculitis (p=0.011) and take a tumor necrosis factor alpha (TNF-α) inhibitor (p=0.045). Female patients were more likely to have neurological involvement (p=0.029).

Conclusion

Awareness of BD symptoms and early recognition can help provide timely and effective treatment to avoid disease complications.

## Introduction

Behçet’s disease (BD) is a chronic variable vessel vasculitis characterized by a multisystem inflammatory disorder that manifests with a wide range of mucocutaneous, articular, ocular, central nervous system (CNS), gastrointestinal, pulmonary, and cardiovascular presentations. Oral and genital ulcers have been reported to be the commonest clinical presentation and were previously considered a mandatory criterion for classification [1]. The disease has been reported worldwide with a specific geographic predilection [2]. For example, Turkey has the highest reported prevalence rate, with approximately 400 cases per 100,000 adults [3]. Also, it is most commonly encountered in the Mediterranean and Far-East countries. Meanwhile, it is low in countries away from the Silk Road. For instance, the prevalence in the United Kingdom, Colombia, Spain, and Japan ranges from 0.64, 1.1, and 6.4 to 16 per 100,000 persons, respectively [4-6]. The etiology and pathogenesis of this disease remain unclear. The mean age at disease presentation is 30-40 years, and it is rarely diagnosed in early and late ages. Moreover, it seems to be underestimated in Black populations [7]. The phenotypic expression also varies among ethnic groups and countries. For example, ocular involvement has been reported to be as high as 60% worldwide; however, it is rarely found in Australian populations [8]. German patients with BD tend to have fewer ocular lesions than Turkish patients [9]. Fewer ocular and genital ulcers have been observed in Chinese patients [10]. In addition, a higher frequency of ocular and neurological manifestations has been reported in Brazil [11]. Gender variations were observed, as genital ulcers and erythema nodosum were found more frequently in women [12]. Two previous cohorts from Saudi Arabia found no significant difference in clinical manifestation or prognosis between male and female patients with BD [13,14]. However, despite decades of disease acknowledgment, large studies addressing BD incidence and prevalence have not been performed. Therefore, this study aimed to analyze and determine the clinical features and characteristics of patients diagnosed with BD in western Saudi Arabia.

## Materials and methods

This single-center study was conducted in a tertiary care center in the western region of Saudi Arabia.

Patients

We included all adult patients aged 14 years or older who were diagnosed with BD according to the International Criteria for Behçet’s Disease (ICBD) at the Department of Rheumatology in National Guards Hospital, western region, Saudi Arabia, from January 2015 to December 2021. The patient’s electronic medical records were reviewed, and their demographic and clinical data were collected by trained rheumatologists. Patients younger than 14 years were excluded from the study.

Data collection

Our data collection sheet included patients’ demographic data, clinical features of BD, and treatment data. The patients’ demographic data included age at the time of symptom onset and diagnosis, sex, BMI, history of smoking, and medical comorbidities. In addition, details of the clinical features of BD were collected, including oral or genital ulcers, skin manifestations, erythema nodosum, uveitis, arthritis, neurological involvement, vascular manifestations, intestinal involvement, and the Pathergy test. We also collected data on the treatment received throughout the disease course.

Statistical analysis

Descriptive statistics are presented as numbers, percentages, means, SDs, and medians (min-max) whenever appropriate. Between comparisons, Fisher’s exact test, independent t-test, or Mann-Whitney U test was applied, and a p-value <0.05 was considered statistically significant. The normality of variables was tested using the Kolmogorov-Smirnov and Shapiro-Wilk tests. Data were analyzed using SPSS version 26 (IBM Corp, Armonk, NY, USA).

Ethics

This study was conducted according to the ethical principles of the Declaration of Helsinki. The study was approved by the Research and Ethics Committee at King Abdullah International Medical Research Center (reference number RJ14/012/J). Data were secured on a safe database. No direct contact with patients was required; therefore, no consent was needed.

## Results

The present study included 91 patients who were diagnosed with BD as per ICBD, with 67 male (73.6%) and 24 female patients (26.4%). The mean age of symptom onset was 29.6 ± 11.4 years; meanwhile, at the time of diagnosis was 31.1 ± 11.9 years. Most patients were overweight, with a mean BMI of 26.7 ± 5.60 kg/m2. The most associated medical comorbidities were diabetes mellitus and hypertension (6.6% for both). The remaining baseline characteristics are presented in Table 1. 

**Table 1:** Baseline characteristics of the patients (n=91).

Study variables	N (%)
Gender	
Male	67 (73.6%)
Female	24 (26.4%)
Smoking	
Yes	16 (17.6%)
No	75 (82.4%)
Associated comorbidities	
None	69 (75.8%)
Diabetes mellitus	6 (6.6%)
Hypertension	6 (6.6%)
Osteoporosis	3 (3.3%)
Fibromyalgia	3 (3.3%)
Chronic lung disease	2 (2.2%)
Cardiomyopathy	2 (2.2%)
Depression	2 (2.2%)
Seizure disorder	2 (2.2%)
Rheumatoid arthritis	1 (1.1%)
Chronic kidney disease	1 (1.1%)
	Mean ± SD
Age at symptoms (years)	29.6 ± 11.4
Age at diagnosis (years)	31.1 ± 11.9
BMI (kg/m2)	26.7 ± 5.60

**Figure 1:**
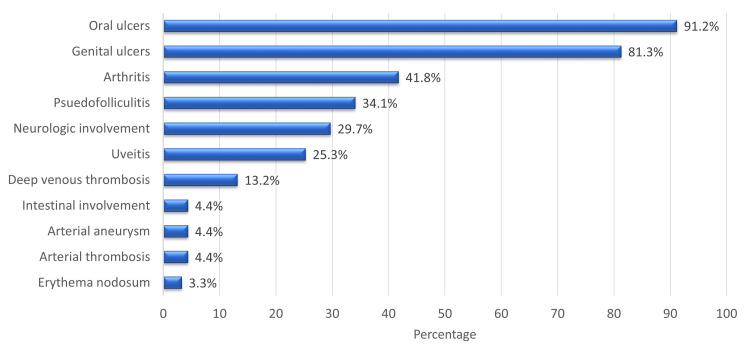
Patients clinical features.

The most common clinical manifestations in our cohort were oral ulcers (91.2%), followed by genital ulcers (81.3%), arthritis (41.8%), and pseudofolliculitis (34.1%). Erythema nodosum was the least commonly reported clinical feature (3.3%) (Figure 1). 

**Figure 2:**
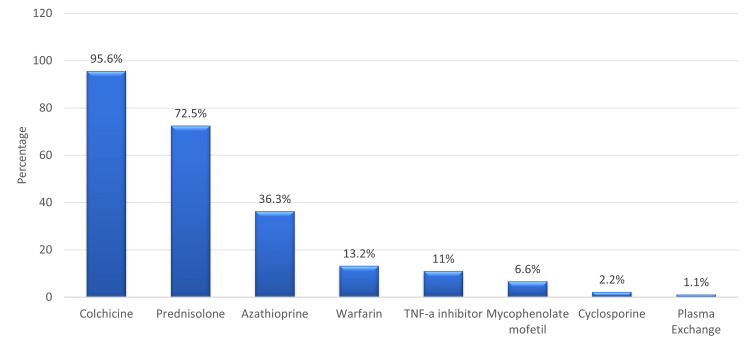
Received medications. TNF-a inhibitor: Tumor necrosis factor-alpha inhibitor.

Colchicine was the most prescribed treatment (95.6%), followed by prednisolone (72.5%) and azathioprine (36.3%) (Figure 2). 

When measuring the differences in the clinical manifestation of BD between male and female patients, it was found that male patients were significantly more likely to be smokers (p=0.005), have pseudofolliculitis (p=0.011), and take a tumor necrosis factor-alpha (TNF-α) inhibitor (p=0.045). Female patients were more likely to have neurological involvement (p=0.029). Additionally, female patients had a significantly higher BMI (p<0.001) (Table 2). 

**Table 2:** Comparison between both sexes (n=91). * Variable with multiple response answers. ** Significant at p<0.05 level.

Factor	Male N (%) (n=67)	Female N (%) (n=24)	P-value
Smoking			
Yes	16 (23.9%)	0	0.005 **
No	51 (76.1%)	24 (10%)	
Associated comorbidities			
Yes	12 (17.9%)	10 (41.7%)	0.027 **
No	55 (82.1%)	14 (58.3%)	
Clinical features *			
Oral ulcers	59 (88.1%)	24 (100%)	0.104
Genital ulcers	55 (82.1%)	19 (79.2%)	0.765
Pseudofolliculitis	28 (41.8%)	3 (12.5%)	0.011 **
Uveitis	19 (28.4%)	4 (16.7%)	0.412
Arthritis	23 (34.3%)	15 (62.5%)	0.029 **
Neurologic involvement	19 (28.4%)	8 (33.3%)	0.795
Deep venous thrombosis	10 (14.9%)	2 (08.3%)	0.506
Arterial thrombosis	4 (06.0%)	0	0.570
Arterial aneurysm	4 (06.0%)	0	0.570
Erythema nodosum	2 (03.0%)	1 (04.2%)	1.000
Intestinal involvement	2 (03.0%)	2 (08.3%)	0.283
Treatment used *			
Prednisolone	48 (71.6%)	18 (75.0%)	1.000
Colchicine	63 (94.0%)	24 (100%)	0.570
Azathioprine	27 (40.3%)	06 (25.0%)	0.222
Tumor Necrosis Factor-alpha inhibitor	10 (14.9%)	0	0.045 **
Mycophenolate mofetil	5 (07.5%)	1 (04.2%)	0.577
Cyclosporine	2 (03.0%)	0	0.392
Warfarin	10 (14.9%)	2 (08.3%)	0.506
Plasma exchange	1 (01.5%)	0	1.000
	Mean ± SD	Mean ± SD	P-value
Age at symptoms onset (years)	28.4 ± 11.1	33.2 ± 11.9	0.072
Age at diagnosis (years)	29.9 ± 11.7	34.2 ± 12.2	0.132
BMI (kg/m2)	25.4 ± 4.73	30.5 ± 6.19	<0.001 **

## Discussion

The current cohort study was conducted in a tertiary care hospital and presented the baseline characteristics and demographics of BD in Saudi Arabia. The mean age of symptoms onset and age of diagnosis was 29.6 ± 11.4 and 31.1 ± 11.9 years, respectively, which is consistent with the first published paper in the region by al-Dalaan AN et al. [13]. A similar finding was observed in data from the Iran Registry, with the mean age of patients being 28.3 ± 8.7 years [15]. However, a later age of onset and diagnosis has been reported in the literature from different geographical areas. For instance, an older paper from the southwestern region of Saudi Arabia showed that the mean age of patients was 37.11 ± 11.90 years [14]. In addition, a multicenter Korean study found that the median age of onset and age at diagnosis was 33 and 41 years, respectively [16]. The mean interval between the time of symptoms onset and diagnosis was approximately two years. Although contrary to other global reports with a low prevalence of disease, this finding may be supported by the awareness of BD among treating physicians in the region. For instance, an eight-year delay between symptom onset and the diagnosis was reported in Switzerland [17].

Interestingly, the prevalence of neurological involvement in BD (neuro-Behçet’s disease) is high in our cohort, reaching 30%, with a statistically significant difference between sexes; however, this finding is less reported in the literature, which could raise the possibility of underdiagnosis of milder forms of BD that do not require medical attention until more severe presentations develop. In the literature, the reported prevalence of neuro-BD is as low as 5.3%, as found by Serdaroglu P et al.; [18] however, higher rates of neuro-Behçet’s presentation were reported within the region in previously mentioned papers as high as 44% and 36.2%, respectively [13,14]. A similar rate was found in a Tunisian study, with a prevalence of 28% [19]. One possible theory explaining the higher prevalence of neuro-BD is that research in a tertiary care center receives referrals from different regional hospitals for further management of more complex cases. With regards to treatment received, colchicine and prednisolone were the most commonly used medications (95.6% and 72.5%, respectively), which was consistent with another study conducted in Tunisia by Daoud F et al. [20]. One patient in our cohort received plasma exchange for refractory neuro-BD with ocular involvement and had a good initial response; however, the loss of follow-up made it impossible to determine the efficacy of such an intervention for such an indication. However, in some case reports, plasma exchange was successfully applied to patients with ocular manifestations. For example, one case describes a 50-year-old lady who presented with bilateral optic neuritis due to BD responding moderately after five sessions of therapeutic plasma exchange [21]. Nevertheless, this is the third-largest cohort of patients with BD in Saudi Arabia. However, the high rate of neurological involvement needs to be addressed in a multicenter study to ascertain the exact prevalence and other clinical manifestations. The study was limited by the loss of outcome assessment, as some patients did not follow-up regularly and were lost to follow-up.

## Conclusions

BD is commonly seen in the western region of Saudi Arabia with different clinical manifestations that align with global reports; however, the prevalence of neuro-BD was high in our study, mainly due to the research being conducted in a high referral tertiary care center. High BMI in females was statistically significantly associated with neuro-BD. Awareness of BD symptoms and early recognition can help provide timely and effective treatment to avoid disease complications.
